# Social Isolation and Mental Health at Primary and Secondary School Entry: A Longitudinal Cohort Study

**DOI:** 10.1016/j.jaac.2014.12.008

**Published:** 2015-03

**Authors:** Timothy Matthews, Andrea Danese, Jasmin Wertz, Antony Ambler, Muireann Kelly, Ashleen Diver, Avshalom Caspi, Terrie E. Moffitt, Louise Arseneault

**Affiliations:** a Medical Research Council (MRC) Social, Genetic and Developmental Psychiatry Centre, Institute of Psychiatry, Psychology and Neuroscience, King’s College London, London; b National and Specialist Child Traumatic Stress and Anxiety Clinic, South London and Maudsley National Health Service (NHS) Foundation Trust, London; c Duke University, Durham, NC, and King's College London

**Keywords:** social isolation, peer relationships, child development, behavioral problems, ADHD

## Abstract

**Objective:**

We tested whether children who are socially isolated early in their schooling develop mental health problems in early adolescence, taking into account their mental health and family risk at school entry.

**Method:**

We used data from the Environmental Risk (E-Risk) Longitudinal Twin Study, a birth cohort of 2,232 children born in England and Wales in 1994 and 1995. We measured social isolation using mothers’ and teachers’ reports at ages 5 and 12 years. We assessed mental health symptoms via mothers’ and teachers’ ratings at age 5 and self-report measures at age 12. We collected mother-reported information about the family environment when children were 5 years old. We conducted regression analyses to test concurrent and longitudinal associations between early family factors, social isolation, and mental health difficulties.

**Results:**

At both primary and secondary school, children who were socially isolated experienced greater mental health difficulties. Children with behavioral problems or attention-deficit/hyperactivity disorder (ADHD) symptoms at age 5 years had an elevated risk of becoming more socially isolated at age 12. However, children who were isolated at age 5 did not have greater mental health symptoms at age 12, over and above pre-existing difficulties.

**Conclusion:**

Although social isolation and mental health problems co-occur in childhood, early isolation does not predict worse mental health problems later on. However, children who exhibit problematic behaviors may struggle to cope with the social challenges that accompany their progression through the early school years.

Intimacy and belongingness are intrinsic human needs.^1^ Interpersonal connections offer many benefits; they provide a frame of reference for social identity, as well as being a source of support and relief in times of stress.^2^ Relationships may be particularly important in childhood, when identity is developing and lifetime trajectories of emotional and behavioral problems are taking shape.^3^, ^4^ To the extent that positive social relationships are rewarding and desirable, the absence or loss of such relationships may be detrimental to individuals’ well-being. Furthermore, children with emotional or behavioral disorders could experience difficulties integrating in social environments. The aim of this study was to examine the associations between social isolation and mental health difficulties at primary and secondary school entry, 2 important transitions in children’s lives and key periods for the formation of social connections.

The majority of studies on social isolation have focused on the latter years of life, when bereavement becomes more common and declining health imposes limitations on social activities. It is also at this stage of life that the long-term impact of social isolation is most evident: isolation in middle to late adulthood is associated with an increased risk of mortality that is comparable to the risks associated with smoking.^5^, ^6^ A number of mechanisms have been proposed to explain how the distressing experience of social isolation negatively affects health, including altered cardiovascular activity,^7^, ^8^ increased activation of the hypothalamic–pituitary–adrenocortical axis,^9^, ^10^ inflammation,^11^, ^12^ and less restorative sleep.^13^, ^14^

Although the health outcomes of social isolation have been extensively studied in adults, there is increasing evidence from longitudinal studies that these long-term effects have their origins much earlier in life. Findings from a birth cohort study in New Zealand show that social isolation measured in childhood is associated with an increased risk of depression, high inflammation levels, and other markers of cardiovascular disease in adulthood.^12^ Furthermore, multiple periods of isolation from childhood to adulthood predict poorer adult health outcomes in a dose–response manner.^15^ This underscores the importance of early intervention to forestall the long-term effects of social isolation, and indicates a need for research to examine this phenomenon from a developmental perspective. Moreover, it is unclear how isolation emerges and persists in childhood. Understanding the role of isolation for children’s development may provide clues as to how it exerts an effect on adult health outcomes.

Research on childhood social isolation has typically focused on the school environment,^16^, ^17^, ^18^, ^19^, ^20^, ^21^, ^22^, ^23^ as it is in this context that children acquire many of their early social experiences and develop peer relationships. This socializing process can be hindered in different ways; for instance, children may experience rejection by classmates, or they may themselves withdraw from social activities, in both cases consigning them to the margins of their peer groups. These isolating experiences may in turn have an impact on mental health. Studies of peer acceptance indicate that rejected children go on to show increases in externalizing problems,^16^, ^19^, ^20^, ^21^ whereas childhood withdrawal is associated with later symptoms of depression and anxiety.^18^, ^24^ However, the relationship between isolation and later psychopathology may not be straightforward. Pre-existing mental health difficulties could play a role, and social isolation may itself be a dynamic phenomenon that co-occurs with poor mental health without necessarily predicting a worsening of mental health symptoms over time. To comprehensively understand the relationship between isolation and mental health, it is important to consider both cross-sectional and bidirectional associations across time.

The onset of adolescence may be particularly challenging for children who did not successfully socialize in their early years of schooling. The transition to secondary school signals a period of upheaval, requiring children to adapt to a larger and unfamiliar social environment. Peer relationships become more complex and nuanced in adolescence,^25^ and solitary behavior could come to be perceived more negatively.^26^ Friendships and interactions with peers provide children with cues from which they can learn etiquette and social norms; therefore, children who are excluded from social interactions in the early years may fail to acquire these skills sufficiently, and go on to display more problematic behavior later in their schooling. However, it is also possible that pre-existing emotional or behavioral problems can alienate children from friendship groups and activities, and thus predict increases in social isolation over time. If this is the case, the association between social isolation and later adjustment outcomes could be largely accounted for by the continuity of these pre-existing problems.

In the present study, we investigated the developmental associations between social isolation and mental health difficulties (emotional problems, behavioral problems, and attention-deficit/hyperactivity disorder [ADHD] symptoms) at ages 5 years and 12 years in a longitudinal, nationally representative cohort of children living in the United Kingdom. First, we tested for concurrent associations between social isolation and mental health difficulties at both ages. Second, we examined bidirectional associations between social isolation and emotional or behavioral problems over time. We controlled for family factors when testing the associations between social isolation and mental health because factors in the family environment such as low socioeconomic status (SES)^27^ and physical maltreatment^28^ could also make children more vulnerable to social isolation.

## Method

### Study Participants

Participants were members of the Environmental Risk (E-Risk) Longitudinal Twin Study, which tracks the development of a birth cohort of 2,232 British children. The sample was drawn from a larger birth register of twins born in England and Wales in 1994 and 1995.^29^ Full details about the sample are reported elsewhere.^30^ Briefly, the E-Risk sample was constructed in 1999 and 2000, when 1,116 families with same-sex 5-year-old twins (93% of those eligible) participated in home visit assessments. Families were recruited to represent the UK population of families with newborns in the 1990s, based on residential location throughout England and Wales, and on mother’s age (i.e., older mothers having twins via assisted reproduction were underselected, and teenage mothers with twins were overselected). We used this sampling to replace high-risk families who were selectively lost to the register via nonresponse and to ensure sufficient numbers of children growing up in high-risk environments. Follow-up home visits were conducted when these children were aged 7 years (98% participation), 10 years (96%), and 12 years (96%). Parents gave informed consent, and children gave assent. Ethical approval was granted by the Joint South London and Maudsley and the Institute of Psychiatry National Health Service (NHS) Ethics Committee.

### Childhood Social Isolation

We measured social isolation using 6 items from the Children’s Behavior Checklist (CBCL)^31^ and the matching 6 items from the Teacher’s Report Form (TRF).^32^ The selected items were “complains of loneliness,” “doesn’t get along with other children [pupils],” “feels or complains that no-one loves him/her,” “would rather be alone than with others,” “not liked by other children [pupils],” and “withdrawn, doesn’t get involved with others.” Mothers completed the questionnaire in a face-to-face interview when children were aged 5 and 12 years, and teachers responded by post. For each respondent, items were summed to create 2 social isolation scales at each age. The mother and teacher scales were moderately correlated both at age 5 years (r = 0.27) and at age 12 (r = 0.31). This level of agreement is consistent with previous findings of parent and teacher ratings of children’s behavior and may be partly accounted for by situational specificity^33^; hence we averaged mothers’ and teachers’ reports to integrate observations both in the classroom environment and outside of school. Cronbach’s α for the combined scale was 0.68 at age 5 years and 0.78 at age 12. To test the genetic contributions to children’s isolation, we used structural equation modeling in OpenMx^34^ to decompose the variance in social isolation into additive genetic, shared environment, and non–shared environment factors.^35^ The best-fitting models indicated that 53% of the variance in social isolation at age 5 years and 41% at age 12 were explained by genetic factors.

We created a categorical social isolation variable to identify 3 groups of children. We classified children with scores of ≤1 as “low” isolated, those with scores >1 and ≤2 as “moderate,” and those with scores >2 as “high.” At age 5 years, 9% of children were highly isolated and 14% were moderately isolated. At age 12 years, 12% of children were highly isolated and 14% moderately isolated. The continuity of social isolation across age points was tested using the κ statistic, indicating slight stability between ages 5 and 12 (κ= 0.17, *p* < .001).

Measures of family factors and mental health problems are described in Table 1. Four of the items used to construct the social isolation measure are included in the emotional problems subscales of the CBCL and TRF; these items were therefore omitted when deriving the emotional problems scale.

### Statistical Analyses

We examined associations between family factors and social isolation using a series of multinomial logistic regressions. We entered family factors into separate univariate regressions, first with social isolation at age 5 and second at age 12 as the dependent variable. As all family factors were significantly associated with social isolation at both age points, we then analyzed them simultaneously in combined models to identify factors independently associated with social isolation.

To test concurrent associations between social isolation and mental health difficulties at ages 5 and 12, we also used multinomial logistic regressions. In the first step, we regressed social isolation on each mental health variable separately (emotional problems, behavioral problems, and ADHD symptoms). In the second step, we entered these variables simultaneously into a single model to test for independent effects. In the final step, we controlled for those family factors that were previously shown to be associated with social isolation at ages 5 and 12 years.

We tested longitudinal associations between age-5 social isolation and age-12 mental health difficulties using linear regressions. For each mental health outcome (depression, anxiety, and conduct problems), we first entered social isolation as the only predictor. In the second step, we controlled for emotional problems, behavioral problems, and ADHD symptoms at age 5 years. In the third step, we controlled for family factors associated with age-5 social isolation. To examine longitudinal associations between age-5 mental health and age-12 social isolation, we used multinomial logistic regressions. In the first step, we entered predictors individually, then in the second step we entered them simultaneously into a combined model. In the third step, we controlled for family factors associated with age-12 social isolation. Finally, in the fourth step, we controlled for prior social isolation.

We did not detect gender interactions for any of the associations investigated. However, because of known gender differences in the prevalence of mental health problems, all analyses were adjusted for gender. Participants in this study were pairs of same-sex twins; hence each family contained data for 2 children. This resulted in non-independent observations, which were adjusted for with tests based on Huber–White or sandwich variance.^36^ These tests adjust estimated standard errors to account for the dependence in the data. We conducted all regression analyses in Stata 11.^37^

## Results

### Association of Early Family Factors and Childhood Social Isolation

Not all family factors were independently associated with social isolation (Table 2). Five-year-old children from low-SES families were at increased risk for being moderately or highly isolated. Children whose mothers had a lifetime diagnosis of depression were at increased risk for high social isolation, whereas those who had experienced physical maltreatment were at increased risk for being moderately but not highly isolated. When looking at age 12, children from lower SES families, children exposed to maltreatment, and children with antisocial parents were at increased risk for being isolated. Children with depressed mothers were not at risk for being socially isolated at age 12. When controlling for baseline social isolation, SES and parental antisocial behavior continued to predict age-12 social isolation, but maltreatment did not.

### Concurrent Association of Social Isolation and Mental Health

Social isolation was concurrently associated with mental health difficulties when children were 5 years of age (Table 3). Children with emotional or behavioral problems or ADHD symptoms all had an increased risk of being moderately or highly isolated. These effects remained significant when controlling for other mental health difficulties and family factors, with the exception of ADHD symptoms, which remained significantly associated with increased risk of high social isolation only.

Social isolation and mental health difficulties were also concurrently associated when children were 12 years of age (Table 3). Children with depression, anxiety, and conduct problems were at increased risk for being isolated. When controlling for other mental health difficulties and age-5 family factors, these effects remained significant except for anxiety, which remained associated with an increased risk of moderate but not high social isolation.

### Social Isolation in Primary School and Mental Health Difficulties at Secondary School Entry

Age-5 social isolation failed to predict age-12 mental health difficulties once baseline mental health problems were taken into account (Table 4). In the first step, children who were moderately and highly isolated reported increased symptoms of depression, anxiety, and conduct problems at age 12. However, these effects did not remain significant after controlling for age-5 emotional and behavioral problems and ADHD symptoms.

### Mental Health Difficulties in Primary School and Social Isolation in Secondary School

Mental health difficulties at age 5 years were associated with higher levels of social isolation at age 12 years (Table 4). Children with behavioral problems or ADHD symptoms at age 5 were at greater risk for being isolated at age 12 than children without mental health problems. These effects remained significant when controlling for family factors and social isolation at age 5 years. Emotional problems at age 5 did not remain significantly associated with social isolation after controlling for baseline isolation.

## Discussion

The present study shows that social isolation is associated with mental health problems in both primary and secondary school-aged children. Furthermore, children who exhibit problematic behaviors such as aggression or hyperactivity in the early school years are at risk for experiencing increasing levels of isolation by the start of secondary school. Children with early emotional problems, on the other hand, do not appear to be at greater risk for increased isolation later on. Mental health symptoms reported by secondary school children who had experienced earlier social isolation are partly accounted for by the continuity of pre-existing problems.

Our first key finding indicates that a number of early family factors are associated with social isolation, both concurrently and across time. Children from low-SES families were more isolated, as were children of antisocial parents. These findings are consistent with previous research on predictors of social isolation.^27^, ^28^ Financial hardship may limit children’s access to social activities, depriving them of opportunities to integrate with their peers. Antisocial behavior on the part of parents, meanwhile, may lead to the family being ostracized by the local community. The children of antisocial parents could also go on to exhibit more behavioral problems themselves and place them at greater risk of becoming isolated. A stable family environment may not only be protective against the risk of social isolation, but may also support the social development of children who are isolated by their peers.^38^

Our second key finding shows that children with early behavioral problems or symptoms of ADHD were at greater risk for becoming more isolated over time than children without these problems. This is consistent with previous findings that aggressive children tend to be rejected by their peers.^17^, ^22^ Prosocial behavior and minimal conflict are valued qualities in children’s friendships.^39^ Therefore, children who are disruptive or aggressive may become excluded from social circles. Interestingly, ADHD symptoms were the strongest predictor of social isolation, a finding that may have important implications for parents, teachers, and practitioners with respect to the early identification and treatment of this disorder. Previous research on children with ADHD suggests that difficulties with self-monitoring and cue-taking can hamper their social interactions, which may contribute to their risk of isolation.^40^ In contrast, although children with emotional problems tend to be socially isolated in primary and secondary school, they did not show increases in social isolation over time.

A third key finding indicates that children who were socially isolated at age 5 years did not show greater depression, anxiety, or conduct problems at age 12 once pre-existing mental health problems were controlled for. This implies that being socially isolated early in life may be an adverse situation that is subsequent to children’s ongoing mental health problems, rather than being a unique precursor to psychopathology. This is surprising, given previous studies showing poor mental health outcomes in rejected and withdrawn children.^16^, ^18^, ^19^, ^20^, ^21^, ^23^, ^24^ A possible explanation for this discrepancy is that the models used in this study controlled more comprehensively for comorbid mental health problems. It may be that the association between social isolation and mental health outcomes is explained by other psychiatric symptoms, or by heterotypic continuity between behavioral and later emotional problems.^41^, ^42^ Another potential explanation is that social isolation was measured differently in this study compared to several prior investigations: rather than focusing specifically on rejection or withdrawal, the items used for our measure of social isolation were broader in scope, comprising a range of difficulties including rejection, peer problems, and social anxiety. Furthermore, we used both mother and teacher ratings, meaning that unlike many prior studies of social isolation, our assessments were not confined to behavior observed during school hours.

An additional explanation for the observed results is that the association between social isolation and mental health may depend on children’s own perceptions of their social relationships. Social isolation and loneliness are related but distinct constructs, one being an objective state and the other a subjective, psychological state.^43^, ^44^, ^45^ It may be that feelings of loneliness are a stronger predictor of mental health outcomes than is objective social isolation.^46^ Longitudinal data on loneliness was not available for this study; nonetheless, it would be advantageous for further research on childhood social isolation to incorporate self-report data as well as teacher, parent, or peer ratings.

A number of limitations to this study warrant acknowledgement. First, the correlational design prohibits any inferences of causality. The etiological association between social isolation and mental health problems is complex and warrants further investigation. Second, age-5 mental health and age-12 social isolation were both based on mother and teacher reports. It is therefore possible that the associations between these measures were inflated because of shared-method variance. Since the children in this study had different teachers at each age, we repeated the analysis using only teachers’ report of social isolation at age 12 years. This yielded the same pattern of results, with behavioral problems and ADHD uniquely predicting increases in social isolation. Future research could investigate whether the longitudinal associations observed in this study can be replicated using different measures and informants.

Third, the categorization of social isolation into groups was based on an arbitrary choice of cutoff points rather than an established precedent. However, similar findings were observed when the analyses were repeated with social isolation analyzed as a linear scale. Finally, as the sample was drawn from a twin study, each of the participants had a sibling by definition. Children with socially anxious or withdrawn behaviors may be protected from social isolation through their sibling relationships.^47^ It is therefore possible that our twin data may underestimate the effects of social isolation.

Findings from this study suggest a number of avenues for further investigation. Examining childhood and adolescent social isolation and its associations with mental health in later life will help to build a more comprehensive picture of social isolation from a developmental standpoint. Furthermore, measures of physical health in late adolescence and early adulthood may prove useful to determine whether the negative effects of childhood social isolation on later health^12^, ^15^ can be detected as early as the teenage years. Finally, investigating subjective ratings of loneliness will help to reveal whether the relationship between social isolation and mental health is modified by individuals’ own feelings about their social lives.

The present study has identified a number of early factors involved in the emergence of social isolation in children. At both primary and secondary school entry, children who are socially isolated experience greater mental health difficulties than their nonisolated peers. However, social isolation in young children did not appear to modify change in their mental health over time; rather, the later problems experienced by these children appear to reflect the stability of pre-existing difficulties. Taken together, our findings suggest that timely intervention to address behavioral problems and ADHD symptoms in early childhood, while being expedient in its own right, may also yield the benefit of preventing children’s social isolation worsening later in their school years.

## Figures and Tables

**Table 1:** Measures of Age-5 Family Factors and Age-5 and Age-12 Mental Health

Measure	Informant	Description	Instrument	Sample Distribution M (SD) or %	Reference
Age 5 y					
Family factors					
SES	Parents	3 groups (split by tertile) based on a standardized composite of income, parents’ education, and social class.	N/A	33.2% (low)	^48^
Maternal depression	Mother	Lifetime diagnosis of a major depressive episode.	Diagnostic Interview Schedule; *DSM-IV*	35.0%	49, 50
Parental antisocial behavior	Mother	Lifetime presence of symptoms of conduct disorder and antisocial personality disorder. Children were coded as having an antisocial parent if either parent had 3 or more antisocial personality symptoms.	Young Adult Self-Report; *DSM-IV*	27.6%	50, 51
Physical maltreatment	Mother	Standardized clinical interview. Children were coded as having experienced maltreatment based on mothers’ report of the severity of discipline, her concerns that someone else may have harmed the child, and the interviewer’s rating of the likelihood that the child had been maltreated.	Interview protocol from the Multisite Child Development Project	13.8%	52, 53, 54
Mental health					
Emotional problems	Mother and teacher	Sum of items on withdrawn/depressed and somatic subscales. Total scores standardized and averaged across raters.	CBCL; TRF	0 (1)	31, 32
Behavioral problems	Mother and teacher	Sum of items on delinquency and aggression subscales, summed across raters and standardized.	CBCL; TRF	0 (1)	31, 32
ADHD symptoms	Mother and teacher	Sum of items on inattentive, impulsive, and hyperactivity scales, summed across raters and standardized.	CBCL; TRF	0 (1)	31, 32
Age 12 y					
Mental health					
Depression	Self-report	Total score of 27 items.	CDI	3.11 (5.32)	55, 56
Anxiety	Self-report	Total score of 10 items	MASC	7.62 (3.04)	^57^
Conduct problems	Self-report	Self-report survey administered via computer. Questions were designed to map onto the diagnostic criteria for conduct disorder.	*DSM-IV*	2.46 (2.94)	^50^

Note: Ages are given in years. CBCL = Child Behavior Checklist; CDI = Children’s Depression Inventory; MASC = Multidimensional Anxiety Scale for Children; N/A = not applicable; SES = socioeconomic status. TRF = Teacher’s Report Form.

**Table 2:** Multivariate Analysis of Associations Between Family Factors and Social Isolation at Age 5 and 12 Years

Age-5 Family Factors	Age-5 Social Isolation^a^				Age-12 Social Isolation^a^			
Moderate RRR (95% CI)		High RRR (95% CI)		Moderate RRR (95% CI)		High RRR (95% CI)	
SES								
Moderate	0.93	(0.67, 1.29)	0.98	(0.64, 1.51)	**1.42**	**(1.01, 1.99)**	**1.59**	**(1.07, 2.38)**
Low	**1.45**	**(1.06, 2.00)**	**1.61**	**(1.06, 2.44)**	**1.67**	**(1.17, 2.37)**	**2.40**	**(1.62, 3.56)**
Maternal depression	1.17	(0.89, 1.54)	**1.75**	**(1.25, 2.46)**	1.22	(0.92, 1.60)	1.10	(0.80, 1.51)
Parental antisocial behavior	1.26	(0.93, 1.71)	1.38	(0.95, 2.00)	**1.58**	**(1.17, 2.13)**	**1.45**	**(1.02, 2.05)**
Physical maltreatment	**1.47**	**(1.04, 2.08)**	1.36	(0.90, 2.06)	1.20	(0.85, 1.69)	**1.50**	**(1.01, 2.22)**

Note: All analyses are adjusted for gender. Boldface type indicates significance. RRR = relative risk ratio; SES = socioeconomic status.

a Social isolation base category: low.

**Table 3:** Concurrent Associations Between Social Isolation and Mental Health at Ages 5 and 12 Years, Controlling for Other Mental Health Problems and Family Factors

Age-5 Mental Health Problems	Age-5 Social Isolation^a^			
Moderate RRR (95% CI)		High RRR (95% CI)	
Emotional problems (unadjusted)	**3.81**	**(3.18, 4.57)**	**7.91**	**(6.23, 10.05)**
Adjusted for age-5 behavioral problems and ADHD	**3.33**	**(2.76, 4.03)**	**6.43**	**(5.01, 8.24)**
Adjusted for age-5 behavioral problems, ADHD, and family factors^b^	**3.33**	**(2.75, 4.03)**	**6.53**	**(5.04, 8.46)**
Behavioral problems (unadjusted)	**1.92**	**(1.69, 2.20)**	**2.73**	**(2.34, 3.19)**
Adjusted for age-5 emotional problems and ADHD	**1.51**	**(1.26, 1.82)**	**1.41**	**(1.11, 1.79)**
Adjusted for age-5 emotional problems, ADHD, and family factors^b^	**1.50**	**(1.24, 1.81)**	**1.42**	**(1.12, 1.81)**
ADHD symptoms (unadjusted)	**1.65**	**(1.45, 1.88)**	**2.86**	**(2.45, 3.34)**
Adjusted for age-5 emotional and behavioral problems	1.08	(0.90, 1.30)	**1.89**	**(1.50, 2.39)**
Adjusted for age-5 emotional and behavioral problems and family factors^b^	1.09	(0.91, 1.31)	**1.94**	**(1.54, 2.44)**

Age-12 Mental Health Problems	Age-12 Social Isolation^a^			
Moderate RRR (95% CI)		High RRR (95% CI)	
Depression (unadjusted)	**1.05**	**(1.02, 1.07)**	**1.12**	**(1.10, 1.14)**
Adjusted for age-12 anxiety and conduct problems	**1.03**	**(1.00, 1.05)**	**1.09**	**(1.07, 1.11)**
Adjusted for age-12 anxiety and conduct problems and age-5 family factors^c^	**1.03**	**(1.00, 1.05)**	**1.09**	**(1.07, 1.11)**
Anxiety (unadjusted)	**1.09**	**(1.05, 1.14)**	**1.13**	**(1.07, 1.19)**
Adjusted for age-12 depression and conduct problems	**1.07**	**(1.02, 1.12)**	1.05	(0.99, 1.11)
Adjusted for age-12 depression and conduct problems and age-5 family factors^c^	**1.07**	**(1.02, 1.11)**	1.04	(0.99, 1.10)
Conduct problems (unadjusted)	**1.10**	**(1.05, 1.15)**	**1.19**	**(1.14, 1.24)**
Adjusted for age-12 depression and anxiety	**1.08**	**(1.04, 1.13)**	**1.14**	**(1.09, 1.19)**
Adjusted for age-12 depression and anxiety and age-5 family factors^c^	**1.06**	**(1.01, 1.12)**	**1.12**	**(1.07, 1.17)**

Note: All analyses adjusted for gender. Boldface type indicates significance. ADHD = attention-deficit/hyperactivity disorder; RRR = relative risk ratio.

a Social isolation base category: low.

b Socioeconomic status (SES), maternal depression, and physical maltreatment.

c SES, parental antisocial behavior, and physical maltreatment.

**Table 4:** Longitudinal Associations Between Social Isolation and Mental Health at Ages 5 and 12 Years, Controlling for Other Mental Health Problems and Family Factors

Age-5 Social Isolation^b^	Age-12 Mental Health Problems					
Depression (B, 95% CI)^a^		Anxiety (B, 95% CI)^a^		Conduct Problems (B, 95% CI)^a^	
Moderate (unadjusted)	**1.18**	**(0.39, 1.98)**	**0.45**	**(0.04, 0.85)**	**0.56**	**(0.16, 0.97)**
Adjusted for age-5 emotional and behavioral problems and ADHD	0.49	(–0.24, 1.23)	0.24	(–0.18, 0.66)	0.30	(–0.10, 0.71)
Adjusted for age-5 emotional and behavioral problems, ADHD and family factors^c^	0.50	(–0.24, 1.24)	0.25	(–0.17, 0.67)	0.29	(–0.11, 0.69)
High (unadjusted)	**2.09**	**(0.95, 3.23)**	**0.84**	**(0.31, 1.36)**	**0.50**	**(0.03, 0.98)**
Adjusted for age-5 emotional and behavioral problems and ADHD	0.58	(–0.75, 1.92)	0.35	(–0.24, 0.94)	−0.06	(−0.61, 0.48)
Adjusted for age-5 emotional and behavioral problems, ADHD and family factors^c^	0.64	(−0.70, 1.97)	0.40	(−0.19, 0.98)	−0.02	(−0.57, 0.53)

Age-5 Mental Health Problems	Age-12 Social Isolation^b^			
Moderate RRR (95% CI)		High RRR (95% CI)	
Emotional problems (unadjusted)	**1.47**	**(1.26, 1.72)**	**2.01**	**(1.71, 2.37)**
Adjusted for age-5 behavioral problems and ADHD	**1.25**	**(1.06, 1.48)**	**1.47**	**(1.23, 1.75)**
Adjusted for age-5 behavioral problems, ADHD, and family factors^d^	**1.21**	**(1.02, 1.43)**	**1.42**	**(1.19, 1.69)**
Adjusted for age-5 behavioral problems, ADHD, family factors,^d^ and social isolation	1.12	(0.93, 1.36)	1.13	(0.93, 1.37)
Behavioral problems (unadjusted)	**1.57**	**(1.38, 1.79)**	**2.19**	**(1.92, 2.50)**
Adjusted for age-5 emotional problems and ADHD	**1.31**	**(1.10, 1.56)**	**1.43**	**(1.20, 1.70)**
Adjusted for age-5 emotional problems, ADHD, and family factors^d^	**1.24**	**(1.05, 1.48)**	**1.38**	**(1.16, 1.66)**
Adjusted for age-5 emotional problems, ADHD, family factors,^d^ and social isolation	**1.22**	**(1.02, 1.46)**	**1.31**	**(1.10, 1.57)**
ADHD symptoms (unadjusted)	**1.51**	**(1.33, 1.71)**	**2.29**	**(2.00, 2.63)**
Adjusted for age-5 emotional and behavioral problems	**1.21**	**(1.02, 1.43)**	**1.67**	**(1.39, 2.00)**
Adjusted for age-5 emotional and behavioral problems and family factors^d^	**1.19**	**(1.00, 1.41)**	**1.64**	**(1.37, 1.97)**
Adjusted for age-5 emotional and behavioral problems, family factors,^d^ and social isolation	1.18	(0.99, 1.39)	**1.55**	**(1.29, 1.87)**

Note: All analyses adjusted for gender. Boldface type indicates significance. ADHD = attention-deficit/hyperactivity disorder; RRR = relative risk ratio.

a Regression coefficient (95% CI).

b Social isolation base category: low.

c Socioeconomic status (SES), maternal depression, and physical maltreatment.

d SES, parental antisocial behavior, and physical maltreatment.
